# Fecundability and Serum PBDE Concentrations in Women

**DOI:** 10.1289/ehp.1002283

**Published:** 2010-08

**Authors:** Julie E. Goodman, John A. Biesemeier, Giffe T. Johnson, Casey Harbison, Raymond D. Harbison, Yiliang Zhu, Richard V. Lee, Hanna Silberberg, Marcia Hardy, Todd Stedeford

**Affiliations:** Gradient, Cambridge, Massachusetts, E-mail: jgoodman@gradientcorp.com; Chemtura Corporation, West Lafayette, Indiana;; College of Public Health, University of South Florida, Tampa, Florida; Department of Medicine and Obstetrics, State University of New York, Buffalo, New York; ICL-IP America, Inc., Potomac, Maryland; Health, Safety & Environment, Albemarle Corporation, Baton Rouge, Louisiana

Harley et al. (2010) reported that as serum concentrations of BDE-47, BDE-99, BDE-100, and BDE-153 [polybrominated diphenyl ether (PBDE) congeners] increased, the time to achieve conception also increased (Harley et al. 2010). Although PBDE concentrations in serum were measured only near the end of the second trimester of pregnancy, the authors reported that the association with a longer time to achieve pregnancy was likely causal. This conclusion is inappropriate for the following reasons.

Although PBDEs are persistent, levels are not completely static, and it is not known how much these levels change in an individual over time, or how PBDE levels during the second trimester of pregnancy differ from those before pregnancy. Given that the interquartile ranges (IQRs) of BDE-47, BDE-100, and BDE-153 were quite small (all with the ratio of the 75th percentile to the 25th percentile being < 3.5) and that exposure measurements were taken only once near the end of the second trimester of pregnancy, even a small difference between the measured PBDE level and the actual levels prior to conception could have led to a relatively high degree of exposure measurement error, biasing the results.

Harley et al. (2010) assessed fecundity using the Cox proportional hazards model. There are two major assumptions of this model. First, there is a multiplicative relationship between the hazard function and the log-linear function of covariates. Harley et al. (2010) did not discuss a mode of action by which this could occur. The second assumption is that the impact of each covariate on hazard remains the same during the entire follow-up period, meaning that all covariates must affect risk in the same proportion over time to prevent a biased risk estimate. The authors did not demonstrate that this is likely the case, either for PBDEs or other covariates.

Many factors can affect when or if pregnancy occurs. Among those not evaluated by Harley et al. (2010) are the timing and frequency of sexual intercourse, the number of potential partners, the timing of ovulation, alcohol consumption (e.g., number of drinks per day), smoking (e.g., number of cigarettes per day), drug use and type, stress-related factors, and paternal factors such as health status, chemical exposures, and behavior (e.g., Eggert et al. 2004). All of these factors could have confounded the reported associations.

The analysis of Harley et al. (2010) also suffers from selection bias—that is, they included only women who became pregnant. The authors explained that if PBDEs are associated with decreased fecundability, then exclusion of nonpregnant women who were trying to get pregnant would bias results toward the null. However, they neglected to discuss the possibility that if PBDEs are not associated with decreased fecundability, excluding these women would bias results away from the null. Because this is precisely the hypothesis being tested, making assumptions either way is inappropriate.

Harley et al. (2010) suggested that interviews conducted at the beginning of pregnancy led to a short recall time for time-to-pregnancy information. They cited several articles on recall of time to pregnancy and menstrual cycle characteristics, but they did not demonstrate whether these were applicable to their study subjects. Thus, recall bias could have led to errors in the outcome measure, leading to unreliable results.

Based on the foregoing limitations, we caution readers to consider that the conclusion reached by Harley et al. (2010)—that PBDEs are associated with decreased fecundability—is not based on robust data and therefore may be inappropriate.
